# Histologic evidence of tracheal stenosis directly resulting from SARS-CoV-2 tissue infiltration, a case series

**DOI:** 10.1186/s13019-022-01839-1

**Published:** 2022-05-26

**Authors:** Tyler B. Draeger, Sarah Tedesco, Shahriyour K. Andaz, Vanessa R. Gibson

**Affiliations:** 1 Department of Surgery, Mount Sinai South Nassau, Icahn School of Medicine at Mount Sinai, Oceanside, NY USA; 2 American University of Antigua College of Medicine, Collidge, Antigua and Barbuda; 3 Division of Cardiothoracic Surgery, Department of Surgery, Mount Sinai South Nassau, Icahn School of Medicine at Mount Sinai, 1 Healthy Way, Oceanside, NY 11572 USA

**Keywords:** Tracheal stenosis, Thoracic surgery, SARS-CoV-2, Tracheal resection, Dilatation, Case series

## Abstract

**Background:**

There has been an anecdotal increase in the incidence of tracheal stenosis that has coincided with the SARS-CoV-2 pandemic.

**Case presentation:**

This is a case series in which we report clinical and pathologic findings of two patients who subsequently developed subglottic tracheal stenosis after having been hospitalized with COVID-19 pneumonia. Histopathologic analysis of tissue from these patients shows features consistent with tissue infiltrated with SARS-CoV-2 virus, namely multinucleated syncytial cells with prominent nucleoli.

**Conclusion:**

Our findings directly implicate SARS-CoV-2 in the pathogenesis of tracheal stenosis.

## Background

Tracheal stenosis is a condition which may occur after tracheal injury or in ventilated patients, after prolonged intubation with either an endotracheal tube or tracheostomy tube. Typical management of clinically significant tracheal stenosis includes resection in appropriate surgical candidates versus bronchoscopic dilatation in those who are not suitable for surgical resection [1].

SARS-CoV-2 continues to be prevalent, with up to 20% of patients with COVID-19 pneumonia developing severe disease [2]. Autopsy and surgical specimens of patients infected with SARS-CoV-2 have demonstrated multinucleated syncytial cells with lymphocytic infiltration on pathologic analysis [3].

Here we present two cases of tracheal stenosis in patients with a recent history of COVID-19 pneumonia, one managed with tracheal resection, the other managed with bronchoscopic dilatations which demonstrated similar histology in surgical and biopsy specimens.

## Case presentation

**Fig. 1:**
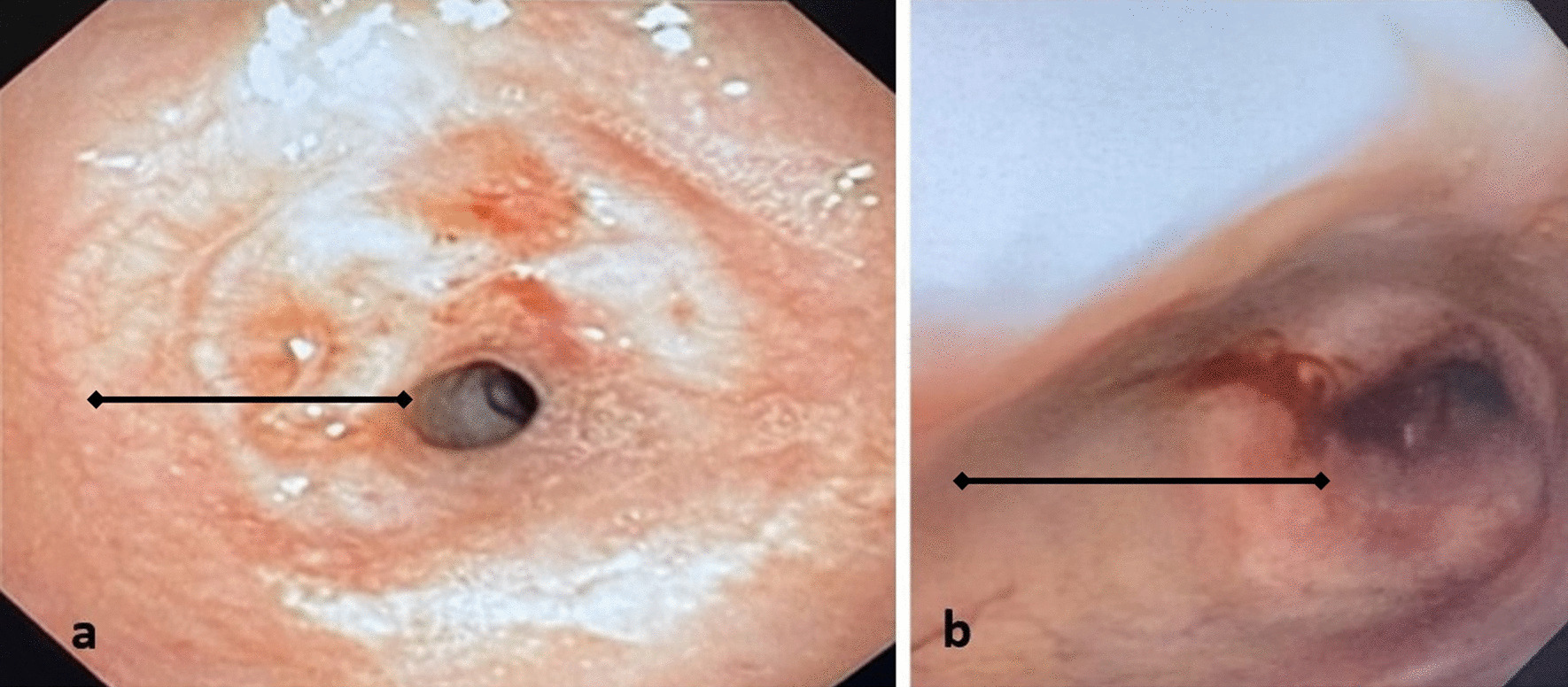
Broncoscopy images of Stenotic Trachea, **a** Case 1, **b** Case 2 (line: region of stenosis)

**Fig. 2:**
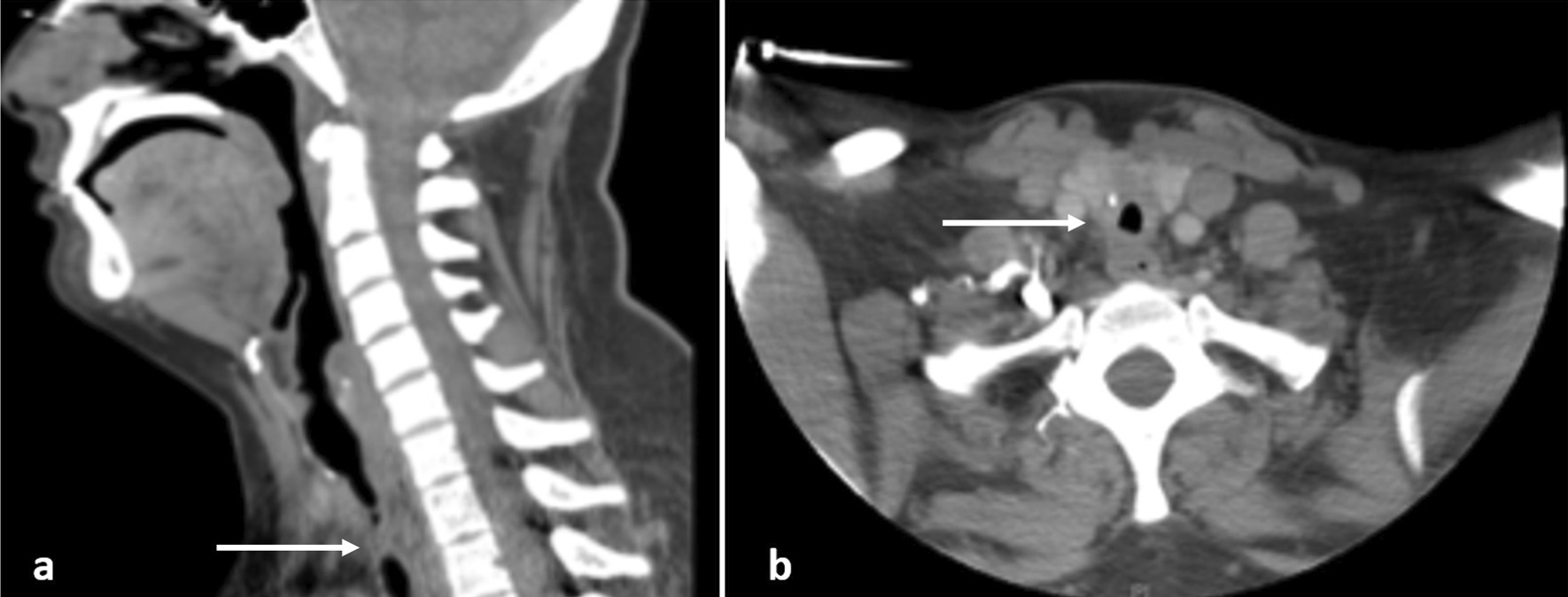
Cross sectional imaging showing tracheal stenosis from Case 1, **a** sagittal, **b** axial (arrow: region of stenosis)

*Case 1* This patient is a 31-year-old female who presented to the emergency department with dyspnea and stridor, worse with exertion. The patient was placed on Bilevel Positive Airway Pressure Ventilation (BiPAP) and arterial blood gas (ABG) showed partial pressure of carbon dioxide (pCO_2_) of 53. The patient had a history of SARS-CoV-2 2 months prior but did not require intubation for COVID-19 pneumonia. She was taken to the operating room for diagnostic bronchoscopy. The trachea appeared 2–3 mm in diameter with extensive circumferential stenosis (Fig. 1a). She underwent balloon dilatation, dilating up to 9 mm until small mucosal fractures were identified. The patient was taken back 3 other times for additional dilatation. She was discharged home with a plan to come back for definitive management. When she returned, she had recurrent stenosis as seen on a Computed Axial Tomography (CT) scan of her neck (Fig. 2); she had repeat bronchoscopy and dilatation and was subsequently taken for tracheal resection.

**Fig. 3:**
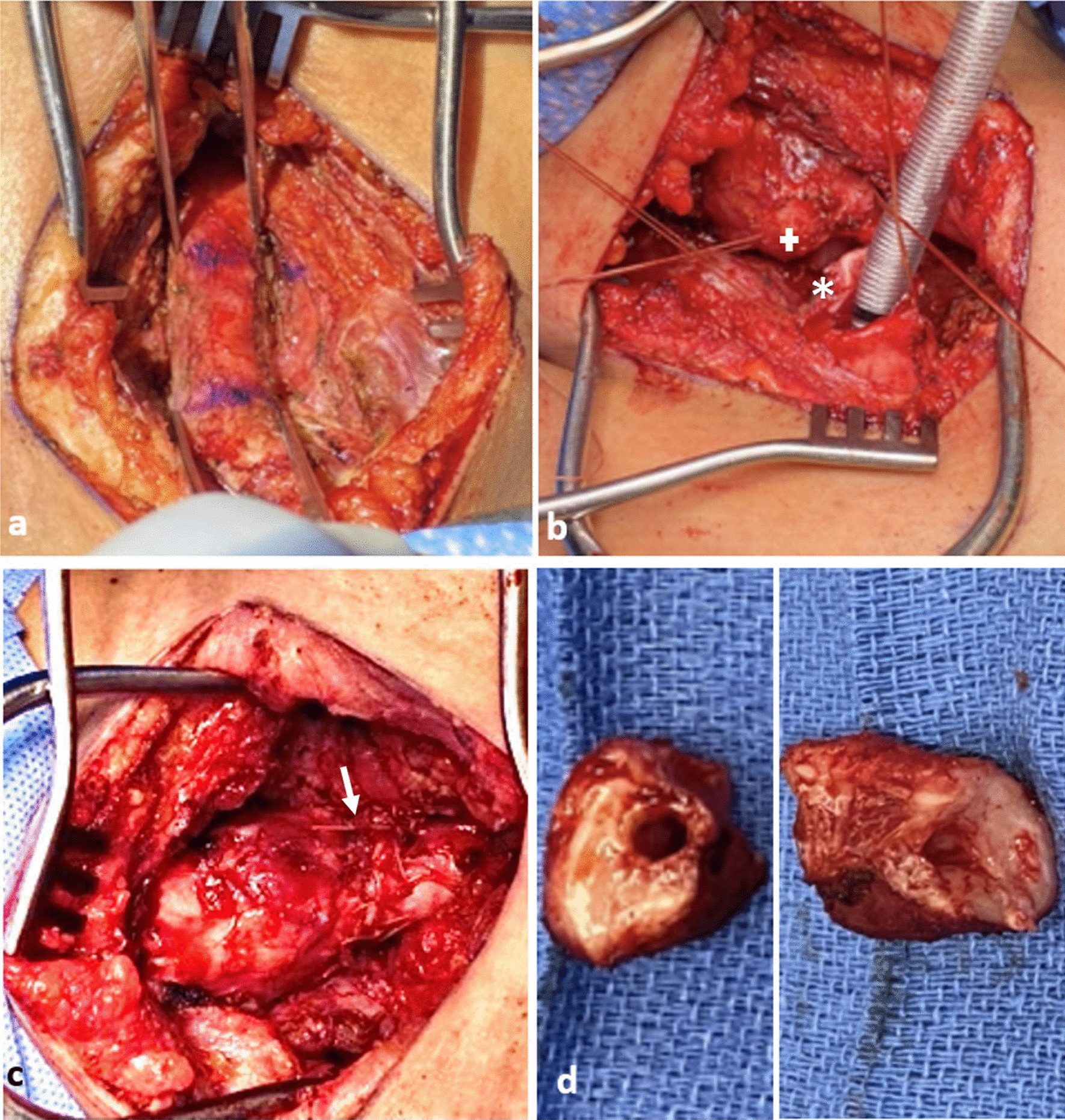
Surgical photographs from Case 1, **a** diseased trachea identified extending from Ring 1–5, **b** diseased trachea is resected (+: proximal, *: distal resection margins), **c** tracheal anastomosis is created (arrow: anastomosis), **d** surgical specimens (proximal and distal rings)

**Fig. 4:**
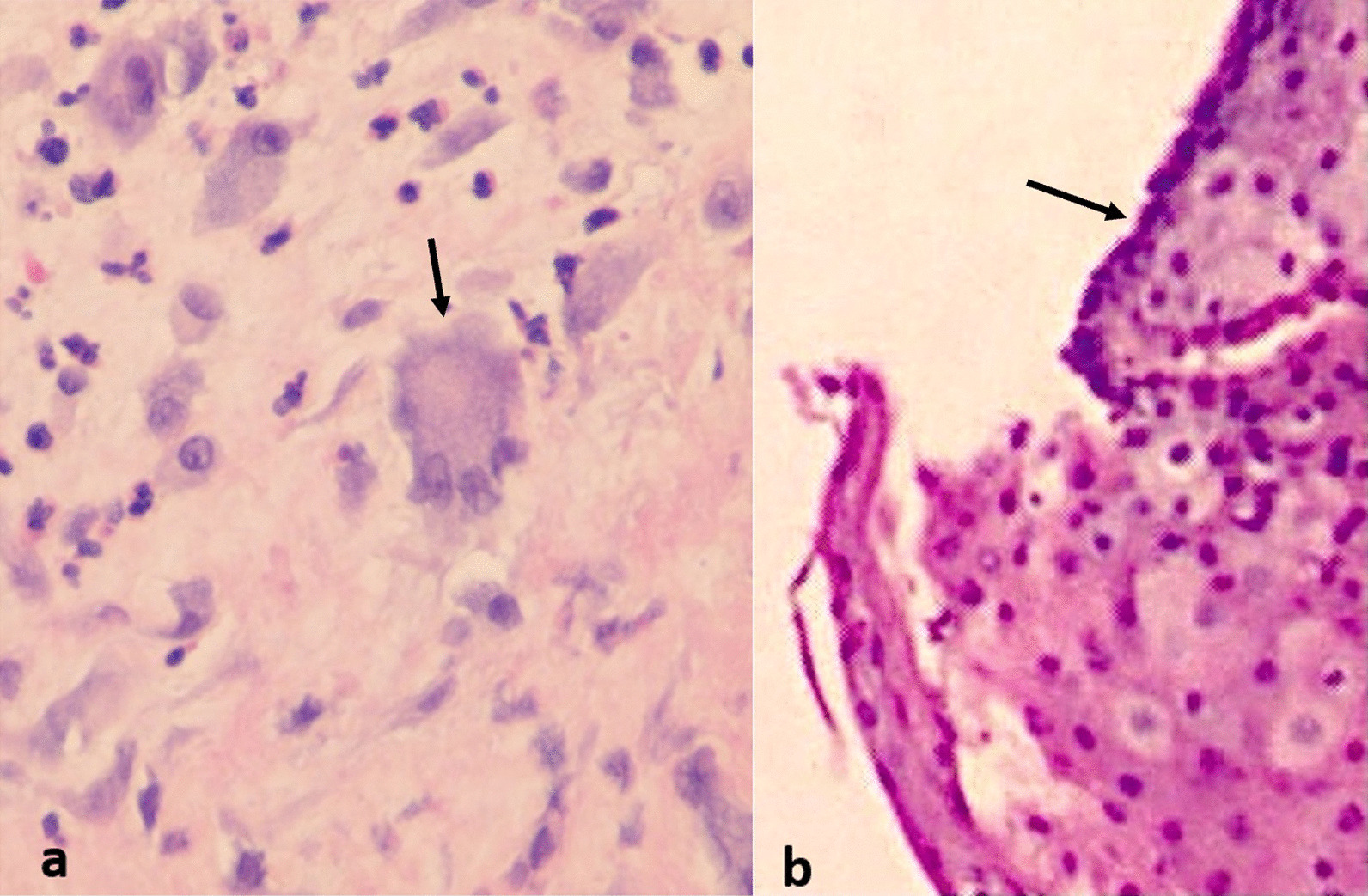
High power micrographs with H&E stain of specimens from Case 1 (**a**) and Case 2 (**b**) (arrows: multinucleated syncytial cells with prominent nucleoli)

The trachea was exposed through a Kocher collar incision; the diseased portion of the trachea extended from ring 1 to ring 5 (Fig. 3a). The proximal and distal trachea was carefully mobilized and the diseased portion of the trachea was resected. An end-to-end anastomosis was performed (Fig. 3b, c). There was significant persistent stenosis in the surgical specimen (Fig. 3d). Pathology revealed multinucleated syncytial cells with lymphocytic infiltration (Fig. 4a). The patient recovered and was discharged home.

*Case 2* This patient is a 45-year-old female who had COVID-19 pneumonia approximately 6 months prior to presentation and required prolonged ventilation. She did require a tracheostomy for this but was subsequently decannulated. She had an extended hospitalization and was subsequently discharged a few weeks prior to presentation. She presented with dyspnea and stridor. She was taken for bronchoscopy and there was circumferential stenosis identified with approximately a 4 mm diameter (Fig. 1b). However, this was proximal to the region of her tracheostomy scar. Bronchoscopic balloon dilatation was performed and the trachea was biopsied. The patient recovered, though required 2 additional dilatation procedures after which the trachea diameter remained stable. The pathology on the biopsy specimen matched that of the tracheal resection from Case 1 (Fig. 4b).

## Discussion

A novel coronavirus was identified in China in late 2019 with similarity to Severe Acute Respiratory Syndrome Coronavirus (SARS-CoV) and was classified as SARS-CoV-2. This virus causes Coronavirus Disease-2019 (COVID-19) pneumonia which may progress to acute respiratory distress syndrome (ARDS) as well as several extrapulmonary manifestations. The spread of SARS-CoV-2 has caused a worldwide pandemic resulting in unmeasurable global devastation.

Since the beginning of the SARS-CoV-2 pandemic, there has been a sharp rise in the number of patients who have required mechanical ventilation. The number of days that patients with COVID-19 pneumonia have required mechanical ventilation has been longer compared to similarly matched patients with other diseases requiring mechanical ventilation, averaging 28.5 days in a Spanish study by Mata-Castro et al. [4]. Patients with COVID-19 pneumonia have required higher positive end expiratory pressures (PEEP) for extended periods of time, which may explain the prolonged duration of intubation in SARS-CoV-2 patients. However, the number of patients with tracheal damage in SARS-CoV-2 infection has been disproportionally high. In a cohort study of 98 patients with COVID-19 pneumonia and severe respiratory failure, Fiaccini et al. [5] observed tracheal damage in nearly half (48%) of COVID-19 pneumonia patients requiring prolonged mechanical ventilation compared to 2% in similarly matched non-SARS-CoV-2 patients.

Acquired tracheal stenosis typically results from trauma or ischemic changes such as prolonged intubation with overinflated balloons, or endoscopic damage to the trachea, with histology showing scar formation and ossific metaplasia [6]. In a study of 20 patients undergoing surgery for tracheal stenosis of benign etiology, Zagalo et al. [7] identified the common findings of hypertrophied submucosa with regions absent of ciliated cells with congestion, hemorrhage, and neovascularization frequently observed, with fibrosis alternating with layers of plasma cell infiltration. Literature review did not identify any published examples of multinucleated syncytial cells as a characteristic of tracheal stenosis in non-SARS-CoV-2 patients.

In the lungs, the viral destruction of pulmonary parenchyma appears to be diffuse alveolar damage (DAD). Xu et al. [3] further showed the presence of multinucleated syncytial cells with prominent nucleoli in specimens from patients with COVID-19 pneumonia. Additional case reports have come to similar conclusions regarding the hallmark histologic characteristics for SARS-CoV-2 infection [2]. Wehzhong and Hualan [8] reported in their recent paper the presumed mechanism of the CaMKII-like system of S protein stimulated membrane fusion by which the syncytia are thought to form. This would indicate that multinucleated syncytial cells are a fundamental histologic characteristic of SARS-CoV-2 infection.

Fiacchini et al. proposed possible etiologies for the increased incidence of tracheal stenosis in patients with COVID-19 pneumonia, citing decreased arterial partial pressure of oxygen to fraction of inspired oxygen (PaO2/FiO2) ratio resulting in hypoxic damage to the trachea, prothrombotic state resulting in microvascular injury and necrosis to the tracheal mucosa, increased use of proning of patients, and high viral replication in the tracheal mucosa [5]. However, our patient in case 1 did not require a period of prolonged intubation which would eliminate most of these other possible etiologies. Given the similarity of the histology on the pathology specimens from case 1 and 2 with that of known SARS-CoV-2 pathologic changes seen in infected tissues, and lack of other possible etiologies for the tracheal stenosis, SARS-CoV-2 may therefore play a direct role in the pathogenesis of the tracheal stenosis in these patients.

## Conclusion

SARS-CoV-2 may play a direct role in the pathogenesis of tracheal stenosis. Further investigation into the etiology of the stenosis is necessary. Biopsy of the trachea during bronchoscopy may show additional patients with multinucleated syncytial cells indicating that SARS-CoV-2 may be implicated in the pathogenesis of the stenosis. As this series involves two patients, more data is required to clarify this link between SARS-CoV-2 and its involvement in the formation tracheal stenosis in this patient population.

## Data Availability

Not applicable.
